# Adaptive evolutionary strategy coupled with an optimized biosynthesis process for the efficient production of pyrroloquinoline quinone from methanol

**DOI:** 10.1186/s13068-023-02261-y

**Published:** 2023-01-19

**Authors:** Yang Ren, Xinwei Yang, Lingtao Ding, Dongfang Liu, Yong Tao, Jianzhong Huang, Chongrong Ke

**Affiliations:** 1 National and Local United Engineering Research Center of Industrial Microbiology and Fermentation Technology, Engineering Research Center of Industrial Microbiology, Ministry of Education, College of Life Sciences, Fujian Normal University, Fuzhou, 350117 Fujian China; 2 CAS Key Laboratory of Microbial Physiological and Metabolic Engineering, Institute of Microbiology, Chinese Academy of Sciences, No. 1 West Beichen Road, Chaoyang District, Beijing, 100101 China

**Keywords:** Pyrroloquinoline quinone, Adaptive laboratory evolution (ALE), *Hyphomicrobium denitrificans*, Response surface methodology (RSM), Two-stage pH control strategy

## Abstract

**Background:**

Pyrroloquinoline quinone (PQQ), a cofactor for bacterial dehydrogenases, is associated with biological processes such as mitochondriogenesis, reproduction, growth, and aging. Due to the extremely high cost of chemical synthesis and low yield of microbial synthesis, the election of effective strains and the development of dynamic fermentation strategies for enhancing PQQ production are meaningful movements to meet the large-scale industrial requirements.

**Results:**

A high-titer PQQ-producing mutant strain, *Hyphomicrobium denitrificans* FJNU-A26, was obtained by integrating ARTP (atmospheric and room‑temperature plasma) mutagenesis, adaptive laboratory evolution and high-throughput screening strategies. Afterward, the systematic optimization of the fermentation medium was conducted using a one-factor-at-a-time strategy and response surface methodology to increase the PQQ concentration from 1.02 to 1.37 g/L. The transcriptional analysis using qRT-PCR revealed that the expression of genes involved in PQQ biosynthesis were significantly upregulated when the ARTP-ALE-derived mutant was applied. Furthermore, a novel two-stage pH control strategy was introduced to address the inconsistent effects of the pH value on cell growth and PQQ production. These combined strategies led to a 148% increase in the PQQ concentration compared with that of the initial strain FJNU-6, reaching 1.52 g/L with a yield of 40.3 mg/g DCW after 144 h of fed-batch fermentation in a 5-L fermenter.

**Conclusion:**

The characteristics above suggest that FJNU-A26 represents an effective candidate as an industrial PQQ producer, and the integrated strategies can be readily extended to other microorganisms for the large-scale production of PQQ.

**Supplementary Information:**

The online version contains supplementary material available at 10.1186/s13068-023-02261-y.

## Introduction

Pyrroloquinoline quinone (PQQ) was first reported as a cofactor for bacterial dehydrogenases [1], of which glucose dehydrogenase [2] and methanol dehydrogenase [3] are the classic examples. PQQ is not currently accepted as a vitamin or conditional vitamin [4], but is regarded as a ‘longevity vitamin’ [5]. Potential health benefits, such as improving antioxidant activity and cognitive functions, enhancing the immune system and mitochondria-related functions, and reducing the level of C-reactive protein, are associated with PQQ supplementation [6–9]. Recently, PQQ was found to effectively alleviate the syndrome of COVID-19 inflammation by protecting cardiopulmonary function and mitochondrial homeostasis in response to hypobaric hypoxia [10]. Moreover, PQQ is produced as a water-soluble PQQ disodium salt (BioPQQ) by fermentation, and its safety has been assessed by the EFSA panel on Dietetic Products, Nutrition, and Allergies (NDA) [11]. In the US, BioPQQ has been filed as a new dietary ingredient (NDI) by the FDA, whereas in the EU, it has been registered as a novel food, enabling its use as a food ingredient. Furthermore, PQQ functions as a biocontrol agent for plant fungal pathogens [12] and a factor promoting plant growth [13]. Therefore, a sufficient supply of PQQ is required due to the extensive applications and growing demands in agricultural, medical, and food and cosmetics areas.

PQQ is widely produced by bacterial species, according to a bioinformatics analysis [14]. Among these species, methanol-utilizing bacteria, such as *Methylobacterium*, *Methylobacillus* and *Hyphomicrobium,* exhibit the highest PQQ yields [15, 16]. In particular, *Hyphomicrobium denitrificans* was used as the only industrial strain to produce PQQ by five biopharmaceutical companies, such as Mitsubishi Gas (Japan) and Hisun Pharmaceutical Co., Ltd. (China). *H. denitrificans* utilizes methanol as its single carbon and energy source through the oxidation of methanol to formaldehyde by methanol dehydrogenase and generates one ATP per methanol molecule through the electron transfer chain [17]. Thus, methanol dehydrogenase is needed in large quantities when *H. denitrificans* grows in the presence of methanol, while PQQ is the cofactor of MDH, which coordinates with Ca^2+^ ions to form Ca^2+^-PQQ moieties and is also needed in large quantities [18]. However, due to the inefficiency or lack of necessary genetic tools, metabolic engineering of methanol-utilizing bacteria remains difficult to achieve [19]. Overexpression of *pqq*ABCDE genes under the control of a truncated *pqq*A2 promoter was conducted successfully only in *Methylovorus* sp. MP688, but the PQQ concentration showed a limited increase (up to ~ 25 μg/ml) [20].

Although the PQQ biosynthetic pathway has recently been elucidated thoroughly [21], metabolic engineering technology was relatively inefficient at obtaining a high PQQ yield due to the unclear molecular mechanism regulating PQQ biosynthesis even in diverse model Gram-negative bacteria. Yang et al. overexpressed the *pqq*ABCDE gene cluster from *Gluconobacter oxydans* in *E. coli* BL21 using the pET28a plasmid, and only 2 mg/L PQQ accumulated in the medium [22]. Sun et al. found that recombinant *K. pneumoniae* harnessing a moderate promoter produced the highest PQQ yield of 0.56 mg/L by trying four distinct promoters with different expression levels to overexpress endogenous PQQ synthetic genes in *K. pneumoniae* along with heterologous expression in *E. coli* [23]. Mi et al. further overexpressed PQQ synthesis-related genes from three repeats of the *tac* promoter in a *K. pneumoniae* strain. After adding sufficient glucose to activate the direct glucose oxidation pathway, 0.78 mg/L PQQ was generated in a 5-L bioreactor [24]. Ye et al. overexpressed the *pqq*ABCDE gene cluster and *tld*D gene under the control of an endogenous constitutive promoter in a *G. oxydans* strain without pyruvate decarboxylase-encoding genes, and a PQQ yield of 51.32 ± 0.8997 mg/L was achieved after the optimization of carbon sources and culture conditions [25]. Wang et al. applied a cell-free in vitro system by incubating the cell-free extract from *G. oxydans* 621H with purified PqqA peptide for PQQ synthesis, and approximately 2.5 mg/mL of PqqA was successfully converted into PQQ with a conversion rate of 70–80% [26]. Considering the yield of PQQ described above, high-yield PQQ strains are relatively difficult to obtain by expressing one or several PQQ synthetic genes through genetic engineering.

Therefore, many fermentation strategies and random mutagenesis approaches have been developed to improve PQQ production in a variety of methanol-utilizing bacteria. Wei PL et al. optimized a culture medium for *Methylobacillus* sp. zju323, and PQQ production reached 232 mg/L in a fed-batch system [27]. Si et al. used a two-stage pH control strategy to increase PQQ production to 353 mg/L in *Methylobacillus* sp. CCTCC M2016079 [28]. Our group developed a two-stage oxygen supply strategy to produce approximately 1 g/L PQQ in *H. denitrificans* FJNU-6 after 140 h in a 5-L fermenter [29]. Thus, optimization of these factors, such as culture medium and fermentation conditions, is effective for increasing the production of PQQ. Meanwhile, random mutagenesis approaches have also been applied to obtain high-yield mutant strains. By integrating ARTP mutagenesis and flow cytometry sorting, a *Methylobacterium extorquens* mutant strain was obtained with 16.02 mg/L PQQ production [30]. The *Methylobacterium* sp. NI91 strain, which was acquired by mutation using UV, NTG, EMS, and LiCl-UV for 11 consecutive generations, showed a 72.44% increase in PQQ yield (19.33 mg/L PQQ) [31, 32]. Among them, ARTP (atmospheric and room‑temperature plasma) is a newly developed whole-cell physical mutagenesis technology that features higher mutation rates than UV radiation or chemical mutagens, especially for organisms with complex metabolic pathways [33].

In the present study, we applied ARTP mutagenesis followed by adaptive laboratory evolution (ALE) and high-throughput screening strategies to obtain a high-titer PQQ mutant strain. Afterward, the optimization of fermentation medium components was conducted using a one-factor-at-a-time strategy and response surface methodology (RSM). The expression of genes involved in PQQ biosynthesis and methanol oxidation was analyzed to elucidate the effects of ARTP mutagenesis and ALE on the initial strain. Moreover, a novel two-stage pH control strategy was developed to further increase the PQQ concentration, productivity and yield in a 5-L fermenter. The integrated strategies described above might be employed to obtain high-titer mutants for the increasing demands in the industrial area of PQQ production.

## Materials and methods

### Microorganism and culture medium

*H. denitrificans* FJNU-6, which was used as the initial strain for PQQ production, was isolated from chemical sewage and preserved by the China General Microbiological Culture Collection Center (Accession No. CGMCC 1.12893). For the screening and evaluation of the high-yield PQQ strains, the screening medium contained 4 g/L (NH_4_)_2_SO_4_, 3 g/L KH_2_PO_4_, 2 g/L Na_2_HPO_4_, 2 g/L MgSO_4_·7H_2_O, and 20–80 g/L CH_3_OH; the initial fermentation medium contained 20 g/L CH_3_OH, 2 g/L (NH_4_)_2_SO_4_, 1.5 g/L KH_2_PO_4_, 3 g/L Na_2_HPO_4_, 1.6 g/L MgSO_4_, 30 mg/L CaCl_2_·2H_2_O, 5 mg/L ZnSO_4_·7H_2_O, 30 mg/L FeSO_4_·7H_2_O, 5 mg/L MnCl_2_·4H_2_O, 3.2 mg/L CoCl_2_·6H_2_O, and 0.5 mg/L CuSO_4_·5H_2_O. The CH_3_OH and (NH_4_)_2_SO_4_ solutions were filtered to remove bacteria, and the other medium components were autoclaved at 121 °C for 20 min.

### ARTP mutagenesis and adaptive directed domestication

The ARTP workflow started with the preparation of 200 μL of the FJNU-6 strain in exponential phase (OD_600_ = 1.0–1.2) followed by dropping the culture onto a sterilized stainless steel plate from an ARTP biological mutagenesis system (ARTP-IIS, Wuxi Yuanqing Tianmu Biological Technology Co., Ltd., Wuxi, China). The operating parameters were as follows: the distance between the plate and the torch nozzle exit was 2 mm, the radio frequency power input was 120 W, the helium gas flow rate was 10 standard liters per minute and the plasma treatment times ranged from 15 to 120 s (15, 30, 45, 60, 75, 90, 105, and 120 s). Subsequently, the treated cells were resuspended in screening medium (2% methanol) and cultivated with the methanol concentration increasing from 2 to 8% at 30 °C for ALE. The colonies of mutants after each ARTP-ALE selection step were collected for rapid screening in fermentation medium of 96-well deep well plates using a SpectraMax microplate reader (i3x; Molecular Devices, LLC, San Jose, CA, USA) by measuring the absorbance at 330 nm (PQQ concentration) and 650 nm (biomass, OD). The strain with the highest PQQ concentration/OD in the former ARTP-ALE round was used for the next ARTP-ALE cycle, and three ARTP-ALE cycles were conducted. Finally, 30 mutant strains with the highest PQQ concentration/OD were isolated from 864 single colonies and then cultured in fermentation medium to detect PQQ production. Among them, ten strains with the highest PQQ production were detected for their stability after 9 consecutive passages.

### Optimization of fermentation medium using one-factor-at-a-time and response surface methodology

Five components of the fermentation medium, including the concentrations of methanol, (NH4)_2_SO_4_, KH_2_PO_4_, Na_2_HPO_4_ and MgSO_4,_ were optimized for PQQ biosynthesis with one-factor-at-a-time approach using the FJNU-A26 strain. Fermentation was conducted in a 250-mL shaker flask, and the five selected medium components were set as follows: methanol concentration (10, 20, 30, 40, and 50 g/L), (NH_4_)_2_SO_4_ (1, 2, 3, 4, and 5 g/L), KH_2_PO_4_ (1, 2, 3, 4, and 5 g/L), Na_2_HPO_4_ (2, 4, 6, 8, and 10 g/L) and MgSO_4_ (0.25, 0.5, 1, 1.5, and 2 g/L). All experiments were performed in triplicate, and the statistical analysis was performed using SPSS software (version 22.0, SPSS Inc., Chicago, IL, USA).

Optimization using response surface methodology (RSM) was also performed in a 250-mL shaker flask, and each of the four factors described above, except for MgSO_4_, was used in three levels according to the results from the single-factor experiments. The respective levels were 10, 20, and 30 g/L for methanol; 1, 2, and 3 g/L for (NH_4_)_2_SO_4_; 1, 2, and 3 g/L for KH_2_PO_4_; and 4, 6, and 8 g/L for Na_2_HPO_4_. The Box–Behnken design (BBD) of RSM designed using Design-Expert software (version 12.0.3.0; Stat-Ease Inc., Minneapolis, MN, USA) was applied to develop a statistical model for establishing the individual and interactive effects of these factors on the fermentation medium components.

### Production of PQQ by fed-batch fermentation in 5-L fermenters

The fermentation of wild and selected mutant strains was performed in 5-L fermentation systems (Shanghai National Center of Bioengineering &Technology, China) containing 2.5 L of fermentation medium optimized based on RSM studies. The optimum fermentation conditions were established as described previously [29] as follows: the temperature was maintained at 30 °C, and a two-stage oxygen supply strategy (60% oxygen supply before 55 h, 40% oxygen supply after 55 h) was used by adjusting the agitator speed, air pressure, and ventilation rate; the pH was maintained at a constant value of 7.0 by streaming NH_4_OH, and methanol was constantly supplied to ensure that its concentration remained at 1–2 g/L in the medium.

The pH value throughout the whole fermentation process was controlled at 6.0, 6.5, 7.0 and 7.5 with the addition of NH_4_OH to establish the two-stage pH control strategy. Other conditions were similar to those from the experiment with the constant pH control described above. For the two-stage pH control strategy experiments, the pH was maintained at 6.5 for the first 40 h and then changed to 7.0 for the rest of fermentation. Samples were collected regularly to analyze biomass, methanol consumption, PQQ concentration and other parameters.

### Assay of gene transcription

Cells from the ARTP-ALE derived strain FJNU-A26 and the initial strain FJNU-6 were collected at 30 h, 42 h, 76 h, 105 h, and 140 h from 5-L fermenters under constant pH 7.0 conditions to extract their total RNA using an RNA plus Kit (Takara Biotechnology, Dalian, China) and then reverse transcribed into cDNAs using a ProtoScript II First-Strand cDNA Synthesis Kit (Invitrogen, Massachusetts, USA). The qRT-PCR assay was conducted using a three-step protocol with the LightCycler® 96 system (Roche, Maryland, USA) and FastStart Essential DNA Green Master reagent (Roche, Maryland, USA), and the primers used in this assay are listed in Additional file 1: Table S1. Two genes, *gapd*H and *rec*A, were used as reference genes, and the target genes transcribed from parallel cDNA samples were assayed 3 times. Finally, gene expression was calculated using LightCycler^®^ Software (version 1.1.0.1320).

### Specific cell growth rate and specific PQQ production rate

The specific cell growth rate and specific PQQ production rate were estimated from experimental or fitted data of cell growth and PQQ production. The values of the specific cell growth rate (*μ*_*x*_) and specific PQQ production rate (*μ*_*p*_) were obtained from the following equations:

$${\mu }_{x}\text{=}\frac{1}{X}*\frac{{\text{d}}X}{{\text{d}}t}{ \mu }_{p} = \frac{1}{X}*\frac{{\text{d}}P}{{\text{d}}t}.$$

The plots of cell growth and PQQ production were fitted to a logistic regression equation (*R*^2^ > 0.99) using Origin Pro 2021 (version 9.8.0.200; Origin Lab Corporation, Northampton, MA, USA), and the parameters *μ*_*x*_ and *μ*_*p*_ were computed from the slope drawn on the plots of semilogarithmic DCW and PQQ concentration versus fermentation time, respectively.

### Analytical methods

The biomass was determined with a UV spectrophotometer (UV1800, Shimadzu, Japan) by measuring the absorbance at 650 nm. The biomass was collected by centrifugation at 8000×*g* and then dried at 105 °C until a constant weight was achieved for the dry cell weight (DCW) determination. High-performance liquid chromatography (Waters e2695) was performed to determine the peak areas of PQQ both in PQQ standard solution and culture media using an XBridge BEH C18 Column (4.6 × 150 mm, 5 μm, Waters) at detection wavelengths of 254 and 330 nm (Additional file 1: Fig. S1). The column temperature was set to 35 °C, the mobile phase was 95:5 water with 1‰ trifluoroacetic acid (TFA):acetonitrile with 1‰ TFA, the flow rate was 1 mL/min, and the injection volume was 10 μL. The PQQ product purchased from Sigma (Sigma-Aldrich-80198) was used as the reference material, and a calibration curve was constructed based on the observed peak areas of PQQ measured by HPLC through a linear regression fitting over the range from 43.9 to 877 mg/L PQQ standard solution with a correlation coefficient of 0.999 (Additional file 1: Fig. S2). Therefore, the PQQ concentration was calculated corresponding to the peak area according to a calibration curve [29, 34]. The concentration of methanol in the medium was analyzed using gas chromatography (Shimadzu, Japan) with a SHIMADZU-Rt-Q-BOND column (30 m × 0.53 mm, DF = 20 μm). Nitrogen was used as the carrier gas, and the initial temperature of the column was set to 110 °C. After holding for 1 min, the gas was heated to 190 °C at a rate of 15 °C/min. Then, the heating rate was changed to 3 °C/min up to 250 °C. The holding time was 4 min, and the injection volume was 1 μL.

## Results and discussion

### Achievement of a high PQQ-producing strain by ARTP mutagenesis combined with ALE

**Fig. 1:**
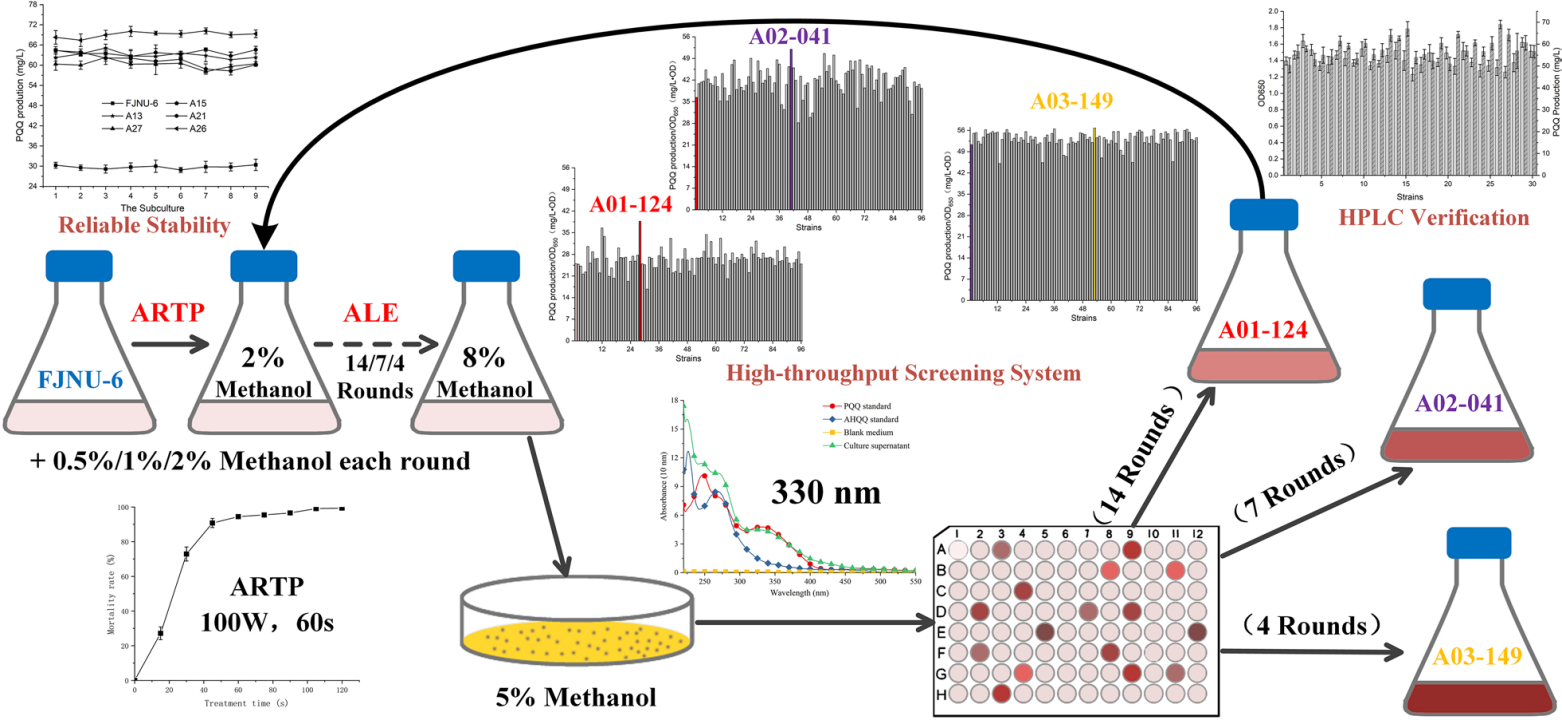
High-yield PQQ-producing strains were achieved using a high-throughput screening system after ARTP mutagenesis with ALE

In our previous study, the wild *H. denitrificans* FJNU-6 strain produced 1.07 g/L PQQ using a two-stage oxygen supply strategy in a 5-L fermenter [29]. A PQQ high-throughput screening system was first established to obtain a high-yield PQQ-producing strain (Fig. 1). For this experiment, a spectrophotometry method using 96-well microtiter plates was developed based on the high correlation coefficient (*R*^2^ > 0.999) between the PQQ content in the culture medium detected using the HPLC method and the spectrometry method measuring the absorbance value at 330 nm [35]. Afterward, *H. denitrificans* FJNU-6 with only 29.6 mg/L PQQ production was subjected to ARTP for three iterative selections, and the exposure time was set at 60 s due to a survival rate of 10%, which is considered appropriate for collecting mutants according to previous reports [36].

After each round of ARTP mutagenesis, a consecutive increase in the methanol concentration was used as the antagonistic factor for adaptive laboratory evolution (ALE). The methanol concentration in the fermentation medium increased from 2% at an interval of 0.5%/1%/2% until the methanol concentration reached 8% during the 1st/2nd/3rd ARTP rounds. ALE is a promising strategy for optimizing strain tolerance to environmental stressors [37]; in particular, another methylotrophic bacterium, *M. extorquens* AM1, with high methanol tolerability exhibited 65% higher mevalonate acid volumetric productivity than that of the control strain in methanol fed-batch fermentation [38]. In our case, ARTP mutagenesis combined with ALE was a process for enriching mutant strains with high-yield PQQ production, and the positive mutation ratio increased from 67.7 to 90.6% after all ARTP-ALE rounds. Finally, 864 single colonies from three iterative selections of ARTP combined with 14/7/4 rounds of ALE were screened using the spectrometry method mentioned above, and 30 mutants with the highest values at A330 nm/OD_650_ were selected for fermentation in shaker flasks to verify their PQQ concentrations (Additional file 1: Fig. S3A). As a result, the mutant strain FJNU-A26 with reliable stability after 9 consecutive passages displayed the highest PQQ production, and the PQQ content reached approximately 69.5 mg/L (Additional file 1: Fig. S3B).

### One-factor-at-a-time and RSM optimization of the fermentation medium

Methanol was reported to promote both cell growth and PQQ accumulation to a greater extent than other carbon sources, such as glucose, glycerol ethanol and fructose, in methanol-utilizing bacteria [15]. Our strain was also identified as a denitrification system [39]. Thus, the presence of relatively high amounts of methanol, NH_4_^+^ and other medium components, such as mineral salts, might affect the biosynthesis of PQQ during fermentation. The concentrations of methanol, (NH_4_)_2_SO_4_, KH_2_PO_4_, Na_2_HPO_4_ and MgSO_4_ were studied for single-factor experiments using the FJNU-A26 strain selected above. After the one-factor-at-a-time optimization, the concentrations of methanol, (NH_4_)_2_SO_4_, KH_2_PO_4_, and Na_2_HPO_4_ were set as 20 g/L, 2 g/L, 2 g/L and 6 g/L, respectively, as the center points for the RSM design (Additional file 1: Fig. S4). For MgSO_4_, no significant changes in PQQ production were observed between 0.25 g and 2 g. According to the results of 29 experimental runs designed by Design-Expert software for the RSM analysis, the observed values for PQQ production varied from 51.2 to 86.5 mg/L in the presence of various levels of four components of fermentation medium (Additional file 1: Table S2). All statistical data, including the *p value* of the quadratic model (< 0.0001), the *p value* of the lack of fit (0.264), and the R-squared and adjusted R-squared values (0.992 and 0.984, respectively) was checked by analysis of variance (ANOVA) (Additional file 1: Table S3), indicating that the equation effectively defined the relationship between PQQ production and each factor.

After fitting the quadratic regression equation, the simulation equation between the four factors and PQQ production was obtained in terms of the coded factors below:

$$ \begin{aligned} Y & =  +  85.112  + 4.58{\text{A }} -  3.39917{\text{B }} +  2.73667{\text{C }} \hfill \\ & \quad -  7.31083{\text{D }} +  7.345{\text{AC}} + 3.135{\text{BC }} +  3.785{\text{CD }} \hfill \\ & \quad -  8.62725{\text{A}}^{2} - 6.031{\text{B}}^{2} -  8.40225{\text{C}}^{2} - 10.926{\text{D}}^{2} . \hfill \\ \end{aligned} $$

**Fig. 2.**
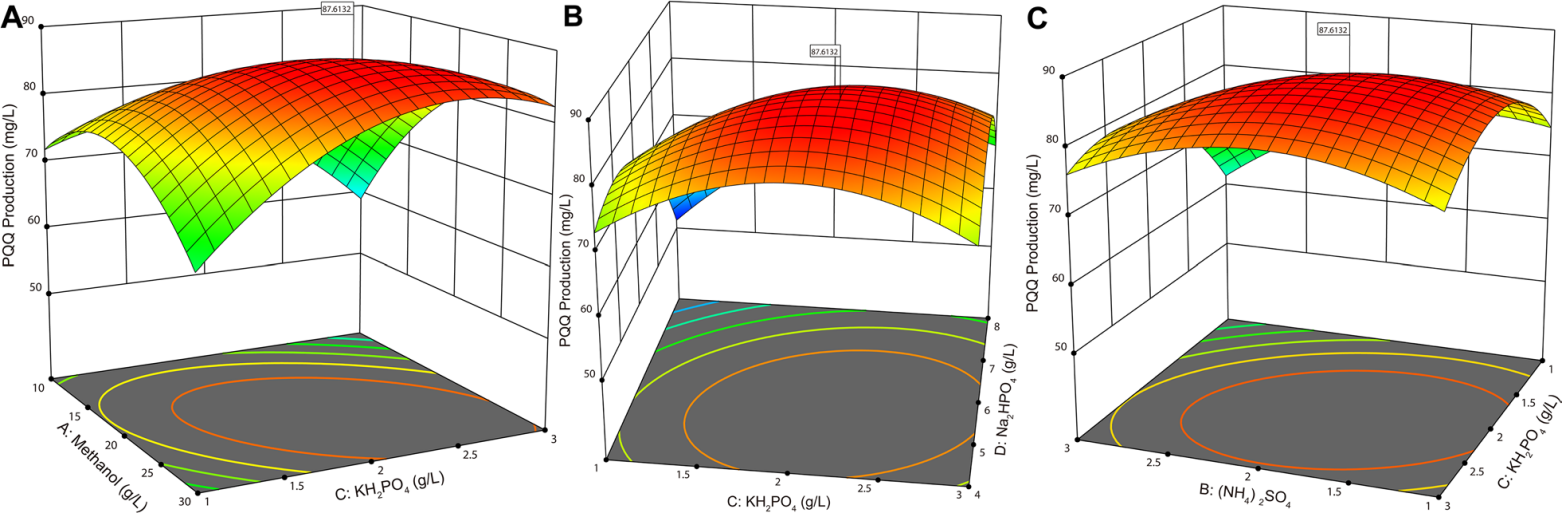
3D response surface plots of interactions on the production of PQQ at *p* < 0.01. **A** 3D surface graph of AC interaction; **B** 3D surface graph of CD interaction; **C** 3D surface graph of BC interaction

In terms of the single factor level, the order of influence is as follows: D (Na_2_HPO_4_) > A (methanol) > B ((NH_4_)_2_SO_4_) > C (KH_2_PO_4_). For the factor interaction, the 3D surface graphs of the significant interaction were generated, and the order was AC > CD > BC (Fig. 2). From the aforementioned analysis, we speculated that sodium concentration exerted the most significant effect over the other factors while potassium concentration showed the strongest interaction with other factors. The maximum PQQ production of 89.1 ± 2.17 mg/L was confirmed (87.6 mg/L for prediction) after the RSM analysis using the following fermentation components: methanol concentration of 23.4 g/L, (NH_4_)_2_SO_4_ concentration of 1.79 g/L, KH_2_PO_4_ concentration of 2.20 g/L and Na_2_HPO_4_ concentration of 5.42 g/L.

### Verification of the ARTP-ALE derived strain with the optimal fermentation media in a 5-L fermenter

**Fig. 3:**
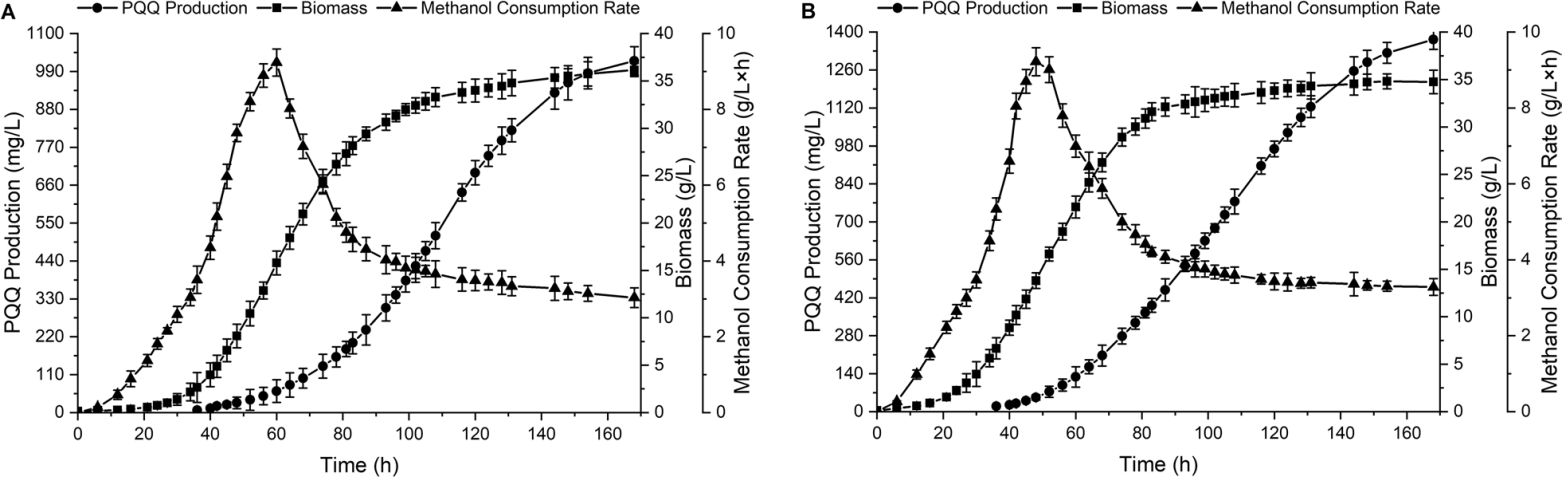
Fed-batch fermentation using a two-stage oxygen supply strategy in a 5-L fermentation tank. **A** Fed-batch fermentation by the wild strain FJNU-6; **B** fed-batch fermentation by the mutant strain FJNU-A26

PQQ production by the selected ARTP-ALE mutant strain FJNU-A26 and the wild strain FJNU-6 were evaluated in a 5-L fermentation tank using the two-stage oxygen supply strategy with the optimized fermentation medium designed above. During fermentation, PQQ started to be synthesized at the point where the speed of methanol consumption peaked and mainly accumulated from the mid-to-late logarithmic growth phase to the stable phase in both strains, which was not completely synchronized with their growth. After 168 h of fermentation, the concentration of PQQ reached 1.37 g/L in the mutant strain, with a maximum specific synthesis rate of 4.91 × 10^–4^ h^−1^ at 105 h (Fig. 3A), while the wild strain produced only 1.02 g/L PQQ, with a maximum specific synthesis rate of 4.69 × 10^–4^ h^−1^ at 116 h under similar conditions (Fig. 3B). Notably, the two strains had comparable methanol consumption tendencies (Fig. 3), and the biomass was also similar in both strains (36.2 g/L of the wild strain vs. 34.7 g/L of mutant strain), indicating that methanol was converted into PQQ more effectively in the mutant strain than in the wild strain. Consequently, after ARTP mutagenesis, ALE and fermentation medium optimization, the PQQ productivity of the mutant strain was 8.17 mg/(L × h), and the PQQ yield was 39.5 mg/g DCW, which was 1.35 times and 1.40 times that of the wild strain with a PQQ productivity of 6.07 mg/(L × h) and a PQQ yield of 28.2 mg/g DCW.

### Analysis of gene transcription to explore the effect of the ARTP-ALE mutant on PQQ biosynthesis

**Fig. 4:**
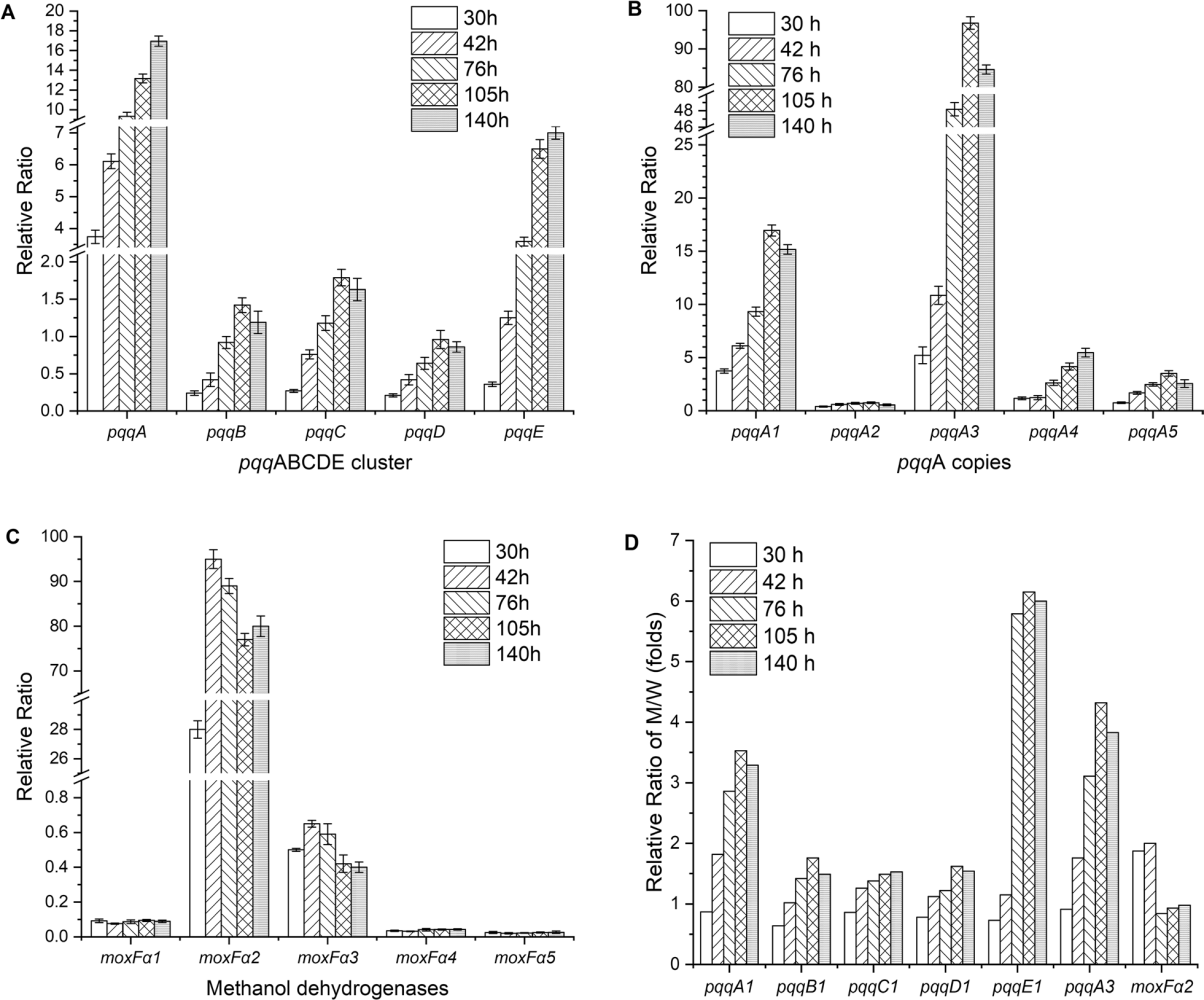
Gene transcription in the ARTP-ALE mutant strain FJNU-A26 and wild strain FJNU-6 in fed-batch fermentation. **A** Expression of the *pqq*ABCDE operon in mutant strain FJNU-A26; **B** expression of five *pqq*A copies in mutant strain FJNU-A26; **C** expression of α subunits of five methanol dehydrogenases in mutant strain FJNU-A26; **D** changes in relative transcription levels between the mutant strain (M) and the wild strain (W)

The transcription of genes involved in methanol oxidation and PQQ biosynthesis was analyzed in both the ARTP-ALE mutant strain FJNU-A26 and the wild strain FJNU-6 to elucidate the effect of the combination of ARTP mutagenesis with ALE on PQQ production and metabolism. Five copies of the *pqq*A genes, which encode products that are the necessary precursors of the PQQ molecule, are present in the genome of our strain. Among them, *pqq*A1 and *pqq*A2 belong to the *pqq*ABCDE and *pqq*ADE operons, while the other three *pqq*A (*pqq*A3-5) genes are located on the genome separately. The qPCR results showed a similar trend for the expression of the *pqq*A1 and *pqq*A3 genes, and these genes were expressed at was much higher levels than other *pqq*A copies (Fig. 4A). Their expression peaked at 105 h, when the specific PQQ synthesis rate of the mutant increased to the greatest extent, which was approximately 3.5 and 4.3 times higher than that of the wild strain, respectively (Fig. 4D). In contrast, the operon *pqq*ADE remained almost completely silent throughout the whole fermentation process in both strains. Notably, the expression of *pqq*E from the operon *pqq*ABCDE in the mutant increased most significantly (P < 0.01) after 42 h when PQQ started to be synthesized and was maintained at approximately 6 times higher levels than that of the wild strain from 76 to 140 h of fermentation (Fig. 4B, D). PqqE controls the cross-linking of Glu-Tyr within PqqA [39], the first step of PQQ synthesis, the high expression of which combined with the high expression of *pqq*A indicated that PQQ biosynthesis was in the active stage. In contrast, the expression of *pqq*B, *pqq*C, and *pqq*D in the mutant slowly (gradually) increased and remained 1 to 2 times higher than that of the wild strain after 42 h of fermentation, indicating that these proteins might be reusable elements, such as PqqD, a peptide chaperone that functions with PqqE [40], or the rate-limiting enzymes involved in hydroxylation (PqqB) [41] and oxidation (PqqC) [42] during PQQ biosynthesis.

The expression of methanol dehydrogenases was also tracked between the two strains. Similar to our previous study [29], *mox*Fα2 played a major role in methanol oxidation because its expression was at least 150-fold higher than the expression of the other four methanol dehydrogenases in both strains. In the mutant, the expression of moxFα2 was increased to the highest value at 42 h of fermentation, after which its expression decreased slightly to approximately 80% up to 140 h of fermentation (Fig. 4C). Compared with that of the wild strain, the expression of moxFα2 in the mutant was approximately 2 times higher before 42 h of fermentation; afterward, the expression of moxFα2 was slightly lower, indicating that the mutant promoted PQQ production instead of the growth of biomass when methanol consumption reached the peak level after 42 h (Fig. 4D).

### Two-stage pH control strategy based on the analysis of *μ*_*x*_ and *μ*_*p*_

The whole fermentation process described above continued for 168 h, and the biomass in both strains slowly increased when fermentation occurred at approximately 90 h, based on the two-stage oxygen supply strategy used. In particular, PQQ continuously accumulated when the unit cell remained stable after the biomass increased to the steady state, suggesting that the acceleration of cell growth might supply a greater labor force to produce PQQ. The pH value became our new focus to reduce the fermentation time and increase PQQ productivity due to its divergent contribution to cell growth and production of many high-value products, such as 5-aminolevulinic acid [43], L-glutamine [44] and eicosapentaenoic acid [45]. The mutant strain FJNU-A26 was cultured in 5-L fermenters with pH values ranging from 6.0 to 7.5 (6, 6.5, 7, and 7.5). As a result, the highest PQQ production and production per unit cell (1.42 ± 0.0481 g/L and 39.0 ± 1.32 mg/g DCW, respectively) were obtained after 168 h of fermentation at pH 7.0. However, the biomass reached a maximum value at 39.6 ± 2.11 g/L when the pH value was maintained at 6.5 after 168 h of fermentation, indicating that specific pH values were beneficial for cell growth or PQQ synthesis (Table 1).

**Table 1:** Effects of the different pH values on PQQ fermentation

pH	Time (h)	Biomass (g/L)	PQQ production (g/L)	PQQ yield (mg/g DCW)	PQQ productivity (mg/L × h)
6.0	168	32.3 ± 1.23	0.908 ± 0.0195	28.1 ± 0.603	5.41 ± 0.116
6.5	168	39.6 ± 1.39	1.22 ± 0.0284	30.8 ± 0.717	7.26 ± 0.169
7.0	168	36.4 ± 2.11	1.42 ± 0.0481	39.0 ± 1.321	8.45 ± 0.286
7.5	168	29.4 ± 0.97	0.920 ± 0.0258	31.3 ± 0.878	5.48 ± 0.154
6.5–7.0	144	37.7 ± 1.26	1.52 ± 0.0348	40.3 ± 0.923	10.5 ± 0.242

**Fig. 5:**
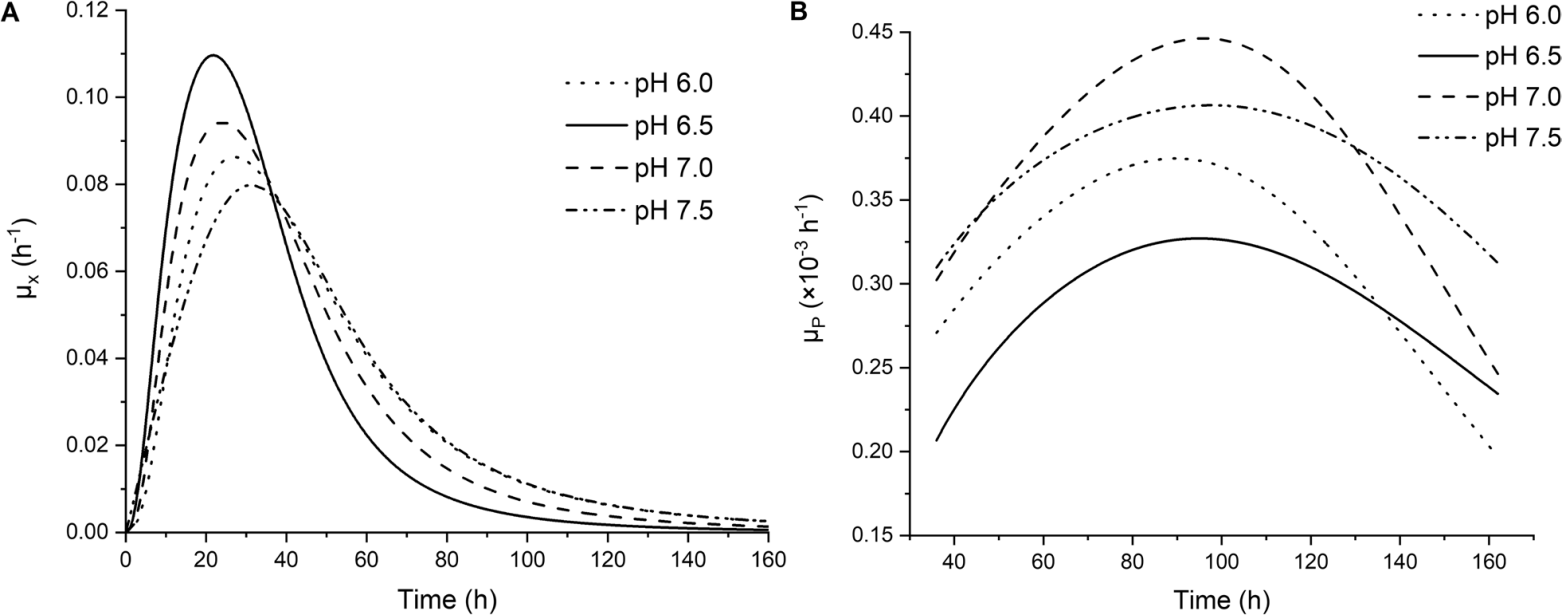
Effects of pH on the specific cell growth rate and the specific PQQ formation rate. **A** Effects of pH on the specific cell growth rate, *μ*_*x*_; **B** effects of pH on the specific PQQ formation rate, *μ*_*p*_

The trends for the effects of pH values on the specific cell growth rate (*μ*_*x*_) and specific PQQ formation rate (*μ*_*p*_) were analyzed to describe the PQQ fermentation process (Fig. 5). The profiles of *μ*_*x*_ and *μ*_*p*_ both fit a normal distribution, and the maximum value of *μ*_*x*_ was obtained at 22 h at a pH value of 6.5, while the maximum value of *μ*_*p*_ was obtained at 100 h at a pH value of 7.0. The explanations for the increase in PQQ biosynthesis by this methylotrophic bacterium at pH 7.0 might be that the *pqq*A promoter was induced, resulting in the upregulation of PQQ precursor [20]. The PqqC protein involved in the oxidation of AHQQ to PQQ may have exhibited higher activity [42], and methanol dehydrogenase functioned better near its optimal pH value [46] when the host was transferred from pH 6.5 to pH 7.0. Noticeably, the *μ*_*x*_ value at pH 6.5 was lower than that at other pH values after 40 h. Therefore, a two-stage pH control strategy was designed to increase PQQ biosynthesis; namely, a pH value of 6.5 favored cell growth and metabolism in the initial fermentation, and a pH value of 7.0 would increase PQQ synthesis after 40 h of fermentation.

### Highly efficient production of PQQ under fed-batch fermentation

**Fig. 6:**
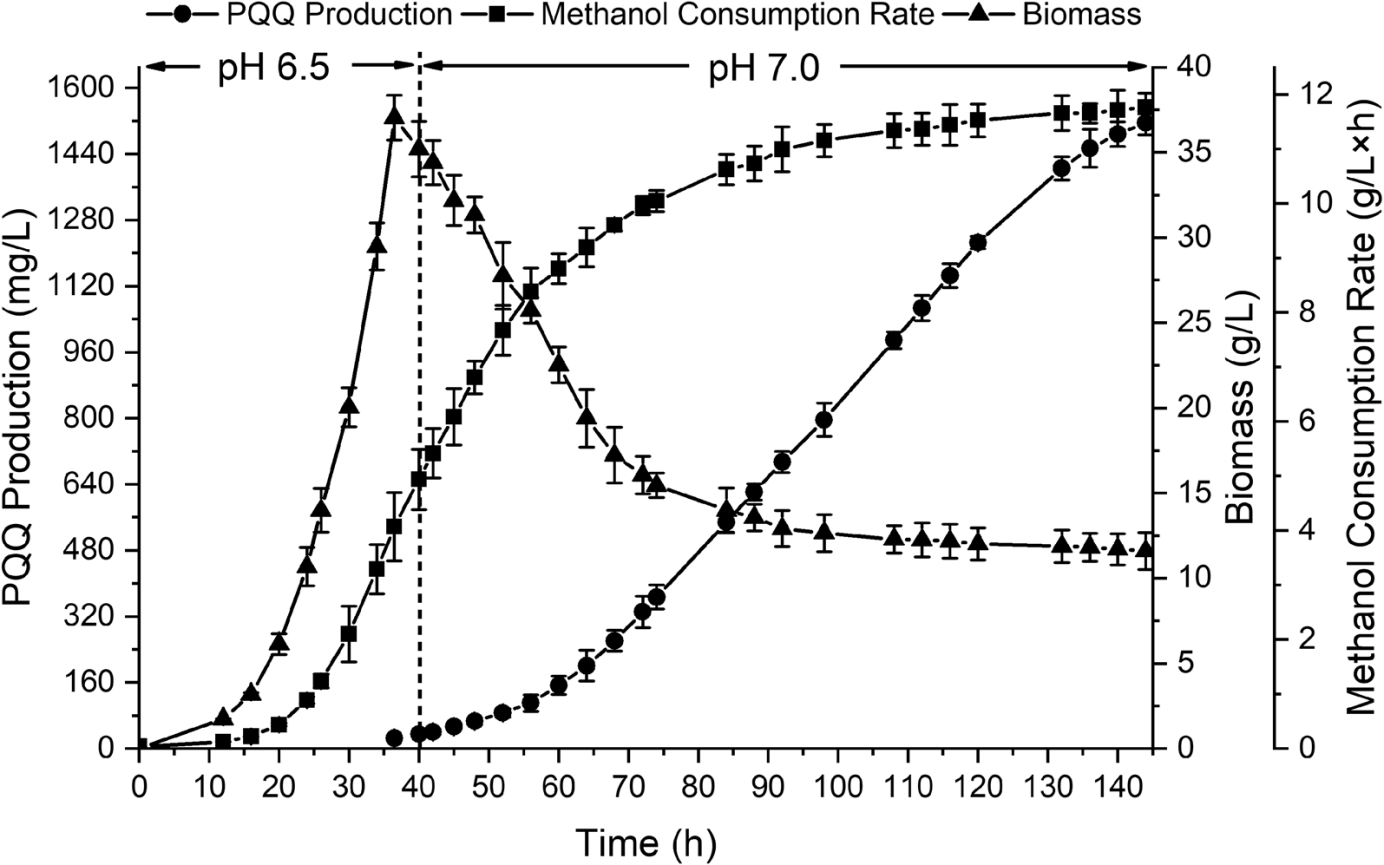
Fed-batch fermentation of mutant FJNU-A26 using an integrated fermentation strategy in a 5-L fermentation tank

The integrated fermentation strategy of the novel two-stage pH control strategy with the previous two-stage oxygen supply strategy was evaluated in a 5-L fermentation tank using the ARTP-ALE derived strain FJNU-A26 (Fig. 6). Compared with the former two-stage oxygen supply strategy, the integrated strategy significantly shortened the fermentation time from 168 to 144 h, when the PQQ content reached its maximum at 1.52 g/L. The PQQ production by bacterial fermentation was a time-consuming process, lasted 6–14 days with a lower productivity at about 1–3 mg/L × h [16, 47]. When constant methanol feeding process and two-phase pH control strategy were applied in 10-L fermentation of *Methylobacillus* sp. CCTCC M2016079, the process was shrunk to 114 h with the PQQ productivity of 3.27 mg/L × h [28]. Our group used adaptive directed domestication and two-stage oxygen supply strategy to further improve the PQQ productivity to above 7 mg/L × h within 6 days [29, 35]. The PQQ productivity here improved up to 10.5 mg/L × h, which was much higher than that of previous strategies.

Meanwhile, the biomass of this integrated strategy reached 37.7 g/L after 144 h of fermentation and became relatively steady after 80 h, which was approximately 10 h faster than that of the constant pH 7.0 strategy. The *μ*_*x*_ value of both strategies reached its maximum at 24 h; however, the *μ*_*x*_ value of the two-stage pH control strategy (0.169 h^−1^) was 1.71-fold higher than that of the constant pH 7.0 strategy. The maximum *μ*_*p*_ of the two-stage pH control strategy reached 5.37 × 10^–4^ h^−1^ at 88 h, and a PQQ yield of 40.3 ± 0.923 mg/g DCW was obtained at the end of fermentation (Table 1). Moreover, higher methanol utilization was detected in the two-stage pH control strategy before 52 h, and the strategy resulted in a greater decrease in the speed of methanol consumption after 36 h of fermentation, which was 16 h shorter than that of the constant pH strategy, reflecting that more methanol was metabolized for fast growth of the mutant strain.

## Conclusion

A highly efficient PQQ producer, *H. denitrificans* FJNU-A26, was obtained through high-throughput screening after ARTP mutagenesis combined with ALE. By optimizing the fermentation medium and introducing a two-stage pH control strategy, the FJNU-A26 mutant showed substantially improved PQQ fermentation performance in fed-batch fermentation, with a higher PQQ concentration (~ 1.52 g/L), productivity (~ 10.5 mg/L × h), yield (~ 40.3 mg/g) and expression of genes involved in PQQ biosynthesis compared with the wild strain. The integrated strategies of ARTP mutagenesis and ALE, as well as optimization of biosynthesis process might provide a meaningful approach to meet large-scale industrial requirements of PQQ.

## Supplementary Information

**Additional file 1: Fig. S1.** HPLC chromatograms of (A) PQQ standard solution (614 mg/L), (B) culture medium after 48 h fermentation and (C) culture medium after 140 h fermentation by adding two volumes of water (diluted three times). **Fig. S2.** The calibration curve of peak areas of PQQ standard solution measured by HPLC vs. different PQQ concentration (877 mg/L, 789 mg/L, 702 mg/L, 614 mg/L, 526 mg/L, 439 mg/L, 351 mg/L, 263 mg/L, 175 mg/L, 87.7 mg/L, 43.9 mg/L). **Fig. S3.** Verification of the ARTP-ALE derived mutant strains isolated from the high-throughput screening. (A) PQQ production and OD_650_ values of thirty ARTP-ALE derived mutant strains with the highest values at A330 nm/OD650 for fermentation in shaker flasks; (B) Genetic stability of five ARTP-ALE derived mutant strains and the wild strain FJNU-6 after nine consecutive passages. **Fig. S4.** One-factor-at-a-time optimization of five factors. (A) methanol; (B) (NH_4_)_2_SO_4_; (C) KH_2_PO_4_; (D) Na_2_HPO_4_; (E) MgSO_4_; The same letter on the bars denote insignificant variations among the levels of the factors (p > 0.05). **Table S1.** Primers used for qRT PCR. **Table S2.** Matrix and results of Response Surface Methodology (RSM) experiments. **Table S3.** ANOVA analysis results for Box–Behnken Design (BBD) experiments.

## Data Availability

All data generated or analyzed during this study are included in this published article and its additional files.
