# Supplementary material for: Adaptive evolutionary strategy coupled with an optimized biosynthesis process for the efficient production of pyrroloquinoline quinone from methanol
Source: Biotechnol Biofuels Bioprod. 2023 Jan 19;16:11. doi: 10.1186/s13068-023-02261-y (PMC9851590; doi:10.1186/s13068-023-02261-y)
Supplement: Supplementary file 1 — Additional file 1: Fig. S1. HPLC chromatograms of (A) PQQ standard solution (614 mg/L), (B) culture medium after 48 h fermentation and (C) culture medium after 140 h fermentation by adding two volumes of water (diluted three times). Fig. S2. The calibration curve of peak areas of PQQ standard solution measured by HPLC vs. different PQQ concentration (877 mg/L, 789 mg/L, 702 mg/L, 614 mg/L, 526 mg/L, 439 mg/L, 351 mg/L, 263 mg/L, 175 mg/L, 87.7 mg/L, 43.9 mg/L). Fig. S3. Verification of the ARTP-ALE derived mutant strains isolated from the high-throughput screening. (A) PQQ production and OD650 values of thirty ARTP-ALE derived mutant strains with the highest values at A330 nm/OD650 for fermentation in shaker flasks; (B) Genetic stability of five ARTP-ALE derived mutant strains and the wild strain FJNU-6 after nine consecutive passages. Fig. S4. One-factor-at-a-time optimization of five factors. (A) methanol; (B) (NH4)2SO4; (C) KH2PO4; (D) Na2HPO4; (E) MgSO4; The same letter on the bars denote insignificant variations among the levels of the factors (p > 0.05). Table S1. Primers used for qRT PCR. Table S2. Matrix and results of Response Surface Methodology (RSM) experiments. Table S3. ANOVA analysis results for Box–Behnken Design (BBD) experiments. [file 13068_2023_2261_MOESM1_ESM.docx]

**ADDITIONAL MATERIALS**

**Adaptive evolutionary strategy coupled with an optimized biosynthesis process for the efficient production of pyrroloquinoline quinone from methanol**

Yang Ren^1†^,; Xinwei Yang^1†^; Lingtao Ding^1^; Dongfang Liu^1^; Yong Tao^1,2^; Jianzhong Huang^1*^; Chongrong Ke^1,2^^[[1]](#footnote-1)^*

^1^ National and Local United Engineering Research Center of Industrial Microbiology and Fermentation Technology; College of Life Sciences, Fujian Normal University, Fuzhou 350117, Fujian, People’s Republic of China

^2^ CAS Key Laboratory of Microbial Physiological and Metabolic Engineering, Institute of Microbiology, Chinese Academy of Sciences, No. 1 West Beichen Road, Chaoyang District, Beijing 100101, China


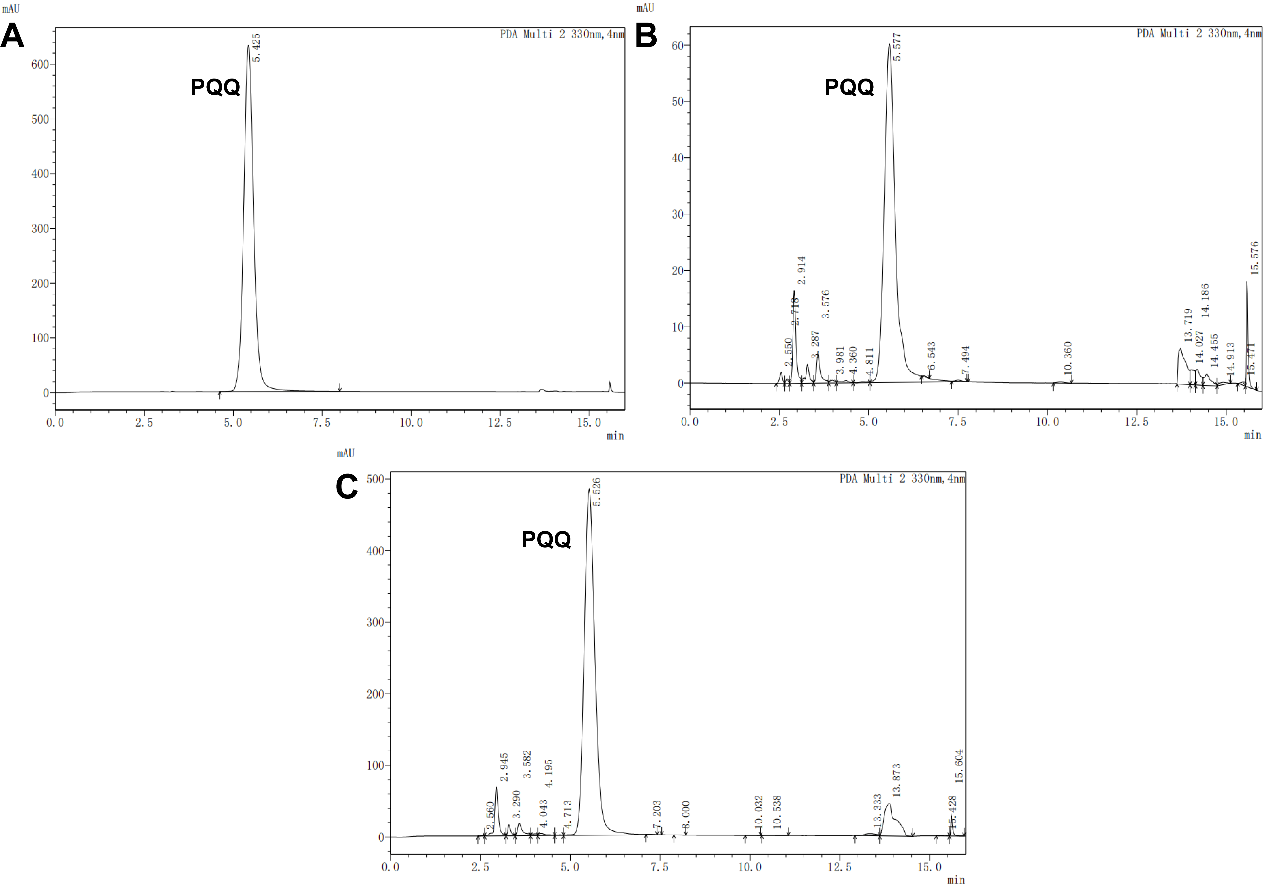


**Fig. S1** HPLC chromatograms of (A) PQQ standard solution (614 mg/L), (B) culture medium after 48 h fermentation and (C) culture medium after 140 h fermentation by adding two volumes of water (diluted three times).





**Fig. S2** The calibration curve of peak areas of PQQ standard solution measured by HPLC vs. different PQQ concentration (877 mg/L, 789 mg/L, 702 mg/L, 614 mg/L, 526 mg/L, 439 mg/L, 351 mg/L, 263 mg/L, 175 mg/L, 87.7 mg/L, 43.9 mg/L).


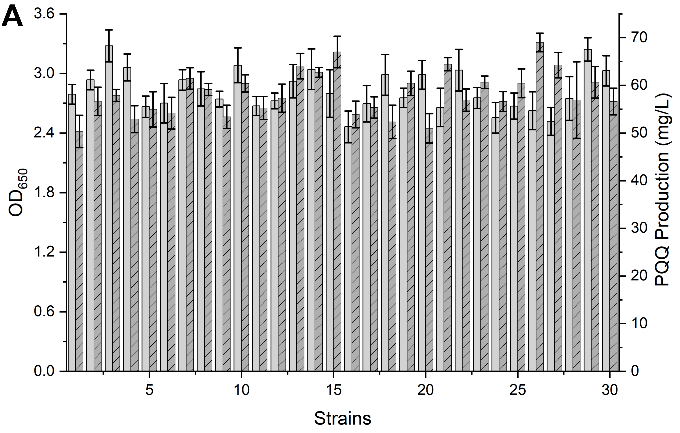

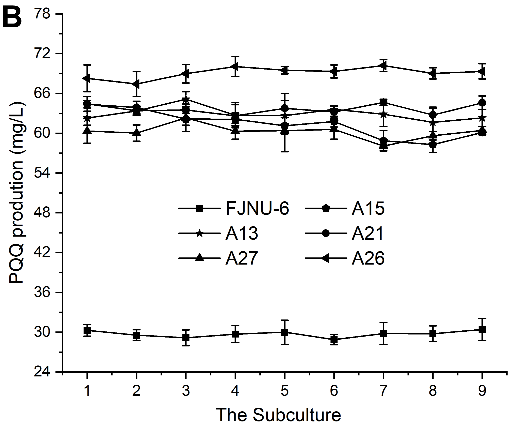


**Fig. S3** Verification of the ARTP-ALE derived mutant strains isolated from the high-throughput screening. (A) PQQ production and OD_650_ values of thirty ARTP-ALE derived mutant strains with the highest values at A330 nm/OD650 for fermentation in shaker flasks; (B) Genetic stability of five ARTP-ALE derived mutant strains and the wild strain FJNU-6 after nine consecutive passages.













**Fig. S4** One-factor-at-a-time optimization of five factors. (A) methanol; (B) (NH_4_)_2_SO_4_; (C) KH_2_PO_4_; (D) Na_2_HPO_4_; (E) MgSO_4_; The same letter on the bars denote insignificant variations among the levels of the factors (p > 0.05).

**Table S1** Primers used for qRT PCR

| Primer ID | Primer sequences(5' - 3') | Description |
| --- | --- | --- |
| *rec*A-F | AGGTCGTCAAGAACAAGG | qRT PCR for *rec*A gene |
| *rec*A-R | CGTTATAGGACATCCAAGC |  |
| *gapd*H-F | CGACTTCAACCACGATGC | qRT PCR for *gapd*H gene |
| *gapd*H-R | TCATACCAGGCGAGAACG |  |
| *pqq*A-F | ATGGAAAGCAGTTACCG | qRT PCR for *pqq*A gene |
| *pqq*A-R | TCAGATGAGGTTGATCTCAG |  |
| *pqq*B-F | ATCGTAAAGCCAGCAAGTC | qRT PCR for *pqq*B gene |
| *pqq*B-R | ATCATCTCGCGGTCTTCG |  |
| *pqq*C-F | AAGGACGGAGCACTCATC | qRT PCR for *pqq*C gene |
| *pqq*C-R | TCATCAGGAAGCAGACCATC |  |
| *pqq*D-F | GCGACGAGTGTTGAGGAAATC | qRT PCR for *pqq*D gene |
| *pqq*D-R | TGTCAGCGAGGATCTCTTGC |  |
| *pqq*E-F | TCGGCATTCTCCAAATCC | qRT PCR for *pqq*E gene |
| *pqq*E-R | GATGAGGTTCGTGTAGAGG |  |
| pqqA2-F | ATGCAAGAGAGCCTGCTT | qRT PCR for *pqq*A2 gene |
| pqqA2-R | CTACCGAACGTAGATGTAGG |  |
| pqqA3-F | ATGAAGACGTGGTCGAAGCC | qRT PCR for *pqq*A3 gene |
| pqqA3-R | TCAGATGACGTCGATTTCAG |  |
| pqqA4-F | ATGAAAGTCTGGACGAAACC | qRT PCR for *pqq*A4 gene |
| pqqA4-R | TTAGATCAGATCGATCTCAGCC |  |
| pqqA5-F | ATGGAGGACATCATGAAGAC | qRT PCR for *pqq*A5 gene |
| pqqA5-R | TTAGATGAGGTCGATCTCGG |  |
| *mox*Fα1-F | CTATGGCAACGAGAAGACGGTGAG | qRT PCR for *mox*Fα1 gene |
| *mox*Fα1-R | GCGGCTGGCTTGACGAATCC |  |
| *mox*Fα2-F | ATCGGGATGCGTCAGAAG | qRT PCR for *mox*Fα2 gene |
| *mox*Fα2-R | CAAGATGAAGTTCGGCTACC |  |
| *mox*Fα3-F | TTGCTGCTGGCTTGACGAAC | qRT PCR for *mox*Fα3 gene |
| *mox*Fα3-R | AGAGCGAAGACGGTGAGTGAG |  |
| *mox*Fα4-F | CGGTATCACAGCACATCAC | qRT PCR for *mox*Fα4 gene |
| *mox*Fα4-R | GTATCTTCACACGCCATTCC |  |
| *mox*Fα5-F | ATCGTAAGCCGTGAGATAGCC | qRT PCR for *mox*Fα5 gene |
| *mox*Fα5-R | ACTGTGGAAGGTGTTGGATGG |  |

**Table S2** Matrix and results of Response Surface Methodology (RSM) experiments.

| Runs | Methanol | (NH_4_)_2_SO_4_ | KH_2_PO_4_ | Na_2_HPO_4_ | PQQ Production | |
| --- | --- | --- | --- | --- | --- | --- |
|  |  |  |  |  | Observed | Predicted |
| 1 | 0 (20) | 0 (2) | 1 (3) | *-1 (4) | 72.39 | 72.05 |
| 2 | 0 (20) | 1 (3) | 1 (3) | 0 (6) | 73.26 | 73.15 |
| 3 | 1 (30) | 0 (2) | *-1 (1) | 0 (6) | 62.46 | 62.58 |
| 4 | *-1 (10) | 0 (2) | 0 (2) | 1 (8) | 54.47 | 52.91 |
| 5 | 1 (30) | 1 (3) | 0 (2) | 0 (6) | 71.26 | 71.73 |
| 6 | 0 (20) | 1 (3) | 0 (2) | 1 (8) | 57.19 | 57.63 |
| 7 | 1 (30) | 0 (2) | 1 (3) | 0 (6) | 83.84 | 82.74 |
| 8 | 0 (20) | 0 (2) | *-1 (1) | *-1 (4) | 75.57 | 74.14 |
| 9 | 0 (20) | 0 (2) | 1 (3) | 1 (8) | 63.15 | 64.99 |
| 10 | 0 (20) | 0 (2) | 0 (2) | 0 (6) | 84.78 | 85.11 |
| 11 | 0 (20) | *-1 (1) | *-1 (1) | 0 (6) | 75.09 | 74.48 |
| 12 | 0 (20) | 0 (2) | 0 (2) | 0 (6) | 86.45 | 85.11 |
| 13 | 0 (20) | 0 (2) | 0 (2) | 0 (6) | 84.06 | 85.11 |
| 14 | 0 (20) | *-1 (1) | 0 (2) | 1 (8) | 63.99 | 64.06 |
| 15 | *-1 (10) | 0 (2) | *-1 (1) | 0 (6) | 66.71 | 68.11 |
| 16 | *-1 (10) | 0 (2) | 0 (2) | *-1 (4) | 68.22 | 69.05 |
| 17 | 1 (30) | 0 (2) | 0 (2) | *-1 (4) | 75.85 | 76.69 |
| 18 | 1 (30) | *-1 (1) | 0 (2) | 0 (6) | 77.12 | 78.34 |
| 19 | *-1 (10) | *-1 (1) | 0 (2) | 0 (6) | 69.42 | 69.37 |
| 20 | 0 (20) | 0 (2) | 0 (2) | 0 (6) | 84.51 | 85.11 |
| 21 | 0 (20) | 1 (3) | *-1 (1) | 0 (6) | 61.65 | 61.41 |
| 22 | *-1 (10) | 1 (3) | 0 (2) | 0 (6) | 63.18 | 62.38 |
| 23 | 0 (20) | *-1 (1) | 0 (2) | *-1 (4) | 79.19 | 79.05 |
| 24 | 1 (30) | 0 (2) | 0 (2) | 1 (8) | 65.14 | 63.59 |
| 25 | 0 (20) | 1 (3) | 0 (2) | *-1 (4) | 71.64 | 71.88 |
| 26 | 0 (20) | *-1 (1) | 1 (3) | 0 (6) | 74.16 | 73.68 |
| 27 | *-1 (10) | 0 (2) | 1 (3) | 0 (6) | 58.71 | 58.89 |
| 28 | 0 (20) | 0 (2) | 0 (2) | 0 (6) | 85.76 | 85.11 |
| 29 | 0 (20) | 0 (2) | *-1 (1) | 1 (8) | 51.19 | 51.95 |

**Table S3** ANOVA analysis results for Box-Behnken Design (BBD) experiemnts.

| Source | Sum of Squares | Degree of freedom | Mean Square | F-value | *p*-value |
| --- | --- | --- | --- | --- | --- |
| **Model** | 2727.49 | 14 | 194.82 | 120.25 | < 0.0001 |
| A-Methanol | 251.72 | 1 | 251.72 | 155.37 | < 0.0001 |
| B-(NH_4_)_2_SO_4_ | 138.65 | 1 | 138.65 | 85.58 | < 0.0001 |
| C-KH_2_PO_4_ | 89.87 | 1 | 89.87 | 55.47 | < 0.0001 |
| D-Na_2_HPO_4_ | 641.38 | 1 | 641.38 | 395.89 | < 0.0001 |
| AB | 0.0361 | 1 | 0.0361 | 0.0223 | 0.8835 |
| AC | 215.80 | 1 | 215.80 | 133.20 | < 0.0001 |
| AD | 2.31 | 1 | 2.31 | 1.43 | 0.2522 |
| BC | 39.31 | 1 | 39.31 | 24.27 | 0.0002 |
| BD | 0.1406 | 1 | 0.1406 | 0.0868 | 0.7726 |
| CD | 57.30 | 1 | 57.30 | 35.37 | < 0.0001 |
| A² | 482.79 | 1 | 482.79 | 298.00 | < 0.0001 |
| B² | 235.93 | 1 | 235.93 | 145.63 | < 0.0001 |
| C² | 457.93 | 1 | 457.93 | 282.66 | < 0.0001 |
| D² | 774.34 | 1 | 774.34 | 477.96 | < 0.0001 |
| **Residual** | 22.68 | 14 | 1.62 |  |  |
| Lack of Fit | 18.89 | 10 | 1.89 | 1.99 | 0.2642 |
| Pure Error | 3.79 | 4 | 0.9474 |  |  |
| **Cor Total** | 2750.17 | 28 |  |  |  |

R^2^ = 0.9918 R^2^_adj_ = 0.9835

1. * Corresponding author at: College of Life Sciences, Fujian Normal University, Fuzhou 350117, Fujian, People’s Republic of China.

   *E-mail address*: kechr@fjnu.edu.cn (ChongRong Ke) and hjz@fjnu.edu.cn (Jianzhong Huang);

   ^†^ Yang Ren and Xinwei Yang contributed equally to this work. [↑](#footnote-ref-1)
